# New Insights into the Design and Application of a Passive Acoustic Monitoring System for the Assessment of the Good Environmental Status in Spanish Marine Waters

**DOI:** 10.3390/s20185353

**Published:** 2020-09-18

**Authors:** Guillermo Lara, Ramón Miralles, Manuel Bou-Cabo, José Antonio Esteban, Víctor Espinosa

**Affiliations:** 1 Institute of Telecommunications and Multimedia Applications (iTEAM), Universitat Politècnica de València (UPV), Camino de Vera S/N, 46022 Valencia, Spain; rmiralle@dcom.upv.es; 2 Unidad Mixta de Investigación IEO-UPV, Tinglados Muelle Frutero, 46370 Grau de Gandia, Valencia, Spain; manuel.bou@ieo.es (M.B.-C.); jose.esteban@hermanosdesal.org (J.A.E.); vespinos@fis.upv.es (V.E.); 3 Instituto Español de Oceanografía (IEO), C.O. Murcia, C/el Varadero 1, 30740 Lo Pagan, Murcia, Spain; 4 Instituto de Inv. para la Gestión Integrada de Zonas Costeras (IGIC), Universitat Politècnica de València (UPV), Campus Gandia, C/ Paranimf 1, 46730 Grau de Gandia, Valencia, Spain

**Keywords:** underwater acoustics, numerical methods, passive acoustic monitoring

## Abstract

Passive acoustic monitoring systems allow for non-invasive monitoring of underwater species and anthropogenic noise. One of these systems has been developed keeping in mind the need to create a user-friendly tool to obtain the ambient noise indicators, while at the same time providing a powerful tool for marine scientists and biologists to progress in studying the effect of human activities on species and ecosystems. The device is based on a low-power processor with ad-hoc electronics, ensuring that the system has efficient energy management, and that the storage capacity is large enough to allow deployments for long periods. An application is presented using data from an acoustic campaign done in 2018 at El Gorguel (Cartagena, Spain). The results show a good agreement between theoretical maps created using AIS data and the ambient noise level indicators measured in the frequency bands of 63 Hz and 125 Hz specified in the directive 11 of the EU Marine Strategy Framework Directive. Using a 2D representation, these ambient noise indicators have enabled repetitive events and daily variations in boat traffic to be identified. The ship noise registered can also be used to track ships by using the acoustic signatures of the engine propellers’ noise.

## 1. Introduction

Assessment of good environmental status (GES) in European seas is at the heart of the Monitoring Guidance for Underwater Noise in European Seas: Monitoring Guidance Specifications [1]. It is essential to monitor and subsequently analyze underwater acoustic data to comply with it properly. There are multiple conditions that cause anthropogenic noise in the marine biota, and passive acoustics monitoring (PAM) allows such sounds to be acquired and continuously studied. To do so, high performance devices must be designed and used properly, following the guidelines set by the Technical Group on Underwater Noise (TGNoise).

It is not easy to monitor Directive 11 on environmental noise in the various marine areas. Indeed, it is essential not only to monitor underwater noise in specific locations, but also to use models that enable noise maps to be estimated. To create these models, many variables need to be known: water column salinity and temperature, marine traffic location, vessel type, speed, etc. The simulations carried out by the models must be checked and corrected using the experimental data obtained with PAM acoustic recorders, and complemented with the information regarding the sources of noise (mostly ships) that it is possible to obtain by analyzing real data.

This manuscript is an extended version of the work published in [2]. In this new paper we describe the most important parts of the electronics and components of the SAMARUC PAM device [3] in greater detail. We also present some new applications as a result of the joint work currently being done by the IEO-UPV mixed research unit for marine studies technologies (UTEM), being the IEO the body in charge of implementing the Marine Strategy for underwater noise in Spain. Using the data acquired by the SAMARUC device, ambient noise indicators are obtained and used to validate the noise map approximations. These maps are created by means of ray theory approximation using the RANDI model [4] to parameterize each ship’s noise source level in the region studied, identified by their Automatic Identification System (AIS) data. For this purpose frequencies 63 Hz and 125 Hz are chosen because they are the ones required by MSFD in the D11.2 to establish a GES in marine waters, and therefore they are the frequencies in which the model must give a correct approximation. This work concludes with an example of how acoustic recordings of ship propeller noise taken with a single broadband hydrophone can be used for noise characterization as well as for target tracking.

## 2. Materials and Methods

### 2.1. SAMARUC as Passive Acoustic Monitoring Device

Working with an autonomous PAM recorder such as the SAMARUC involves several steps: first, the device is deployed for several months in a desired location where it registers acoustic information about the species and anthropogenic noises; after that, the system is recovered, the data extracted from device’s memory and saved as wav files in a local server. Finally, the data is analyzed offline by means of specific software (SAMARUC software is registered by UPV [5]). The SAMARUC acoustic recorder provides high bandwidth submarine audio data at a sampling rate of 192 kHz.

The system’s main component is a general-purpose, ultra-low power consumption digital signal processor (DSP) from Texas Instruments, the TMS320C5535. The selection of the DSP is made as a trade-off among price, low-power consumption, flexibility, and reliability. An adequate selection of the DSP and peripherals allows the time between service intervals to be increased and the battery life extended.

**Figure 1:**
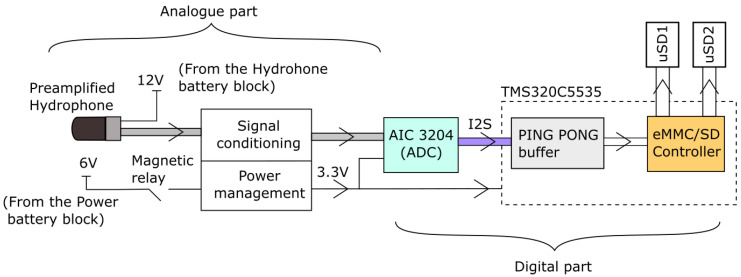
Block diagram of the SAMARUC system. Audio differential signals shaded in grey. I2S bus shaded in purple.

Figure 1 shows how the electronics used work in a block diagram. One can see how the digital data from the low-power analog-to-digital converter (ADC) AIC3204 arrives through the I2S data bus to the PING_PONG buffer. To enhance the signal-to-noise ratio, the power supply and conditioning circuit have been isolated.

The AIC3204 has been set up to perform a BIQUAD filter to establish 192 kHz as a sampling frequency, and it is able to add a gain in order to amplify the signal if it is necessary to increase the sensitivity of the selected hydrophone depending on the marine strategy deployment category. In any case, a system sensitivity (hydrophone + AIC3204 gain) of between −165 and −185 dB re 1 V/uPa is recommended [1].

Table 1 sums up the main specifications of the SAMARUC, many of them obtained by means of the calibration carried out in the IEO-UPV facilities located in the port of Gandía (Valencia, Spain) following the recommendations given in [1]. The choice of hydrophone is one of the most important matters to achieve the desired sensitivity. In our case, we use the Cetacean Research Company’s pre-amplified hydrophone C57.

**Figure 2:**
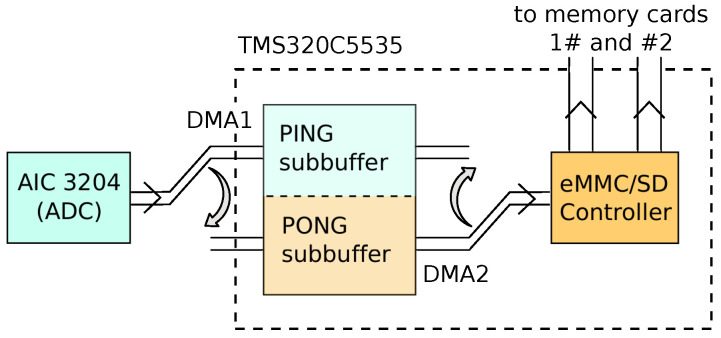
Block diagram detail of the Ping-Pong buffer.

Data transmission is set up in DMA Ping-Pong mode, so the system records acoustic data without sample loss while storing that data in the microSD cards. The Ping-Pong settings allow a buffer (PING_PONG buffer) to be used, which is internally divided into two subbuffers (called subbuffer_PING and subbuffer_PONG), allowing the status of each subbuffer to be checked for the tasks to be carried out in parallel. Thanks to the integration of the two DMAs, simultaneous transmission between memory and peripherals is available. Figure 2 shows these simultaneous tasks. When a DMA (DMA1, for instance) is set up to transfer data from AIC3204 to one subbuffer, DMA2 will send the data from the other to the uSD, alternating these two tasks as the AIC3204 fills the subbuffer where it is working.

#### 2.1.1. Specific Electronics

A dedicated electronic board was designed whose functions include, among others, adding a second microSD card to double the available storage capacity. This electronic design (called signal conditioning in Figure 1) isolates the analog audio signal captured by the hydrophone (including its amplified components) from the digital signals and power supply of the peripherals and processor by adding two independent grounds, reducing the interferences from the processor’s electronics on the low-level hydrophone signal, and increasing the signal-to-noise ratio.

The block called power management in Figure 1 includes components for electromagnetic compatibility (EMC) protection and a regulator (TPS62260DDCR) that lowers the voltage from 6 V to 3.3 V. The regulator can provide a constant voltage of 3.3 V as long as the battery pack voltage is above 2 V. If the battery pack voltage falls below this, the system has a hard stop.

The system has two clocks (not shown in Figure 1): the ASDM1-12.000MHZ-LC-T, which controls the DSP, and one real-time clock, the SSPT7F-12.5PF20-R, which is used to control the duty cycle of the system. Both are low power consumption devices and have good stability over temperature. The real-time clock has a frequency stability of ±20 ppm, which gives a drift of ±10.5 min/year. Typically, upon recovery of the device, a comparison of the internal clock with a precision external GPS reference clock is made. The clock drift is considered for the subsequent signal processing stages such as noise modelling with AIS data and comparison with theoretical sound maps.

#### 2.1.2. File System

Audio data is recorded sequentially in a passive acoustic device, and thus the file allocation table can be greatly simplified. We have taken advantage of this to create a proprietary file system designed for enhanced speed and low latency in read-and-write access.

The formatting process divides the memory into three parts of different sizes: the header, the Block-File allocation table, and the data section. The header is used to set up the device’s operation, and it stores information about the AIC3204’s sampling frequency, recording cycle (minutes ON-OFF) and gain. All this information is loaded on powering up the device. The Block-File allocation table stores information about the block start and block end of each of the files, as well as the time stamp of the first sample. Finally, the data section stores the samples of the recording in the different blocks of the uSD card. The system is put into sleep mode during the OFF cycle to preserve battery life.

#### 2.1.3. Housing and Battery Blocks

A pressure case was built from a tube of 6082 aluminum anodized with NITUFF, measuring 12 × 70 cm (diameter × length). This material was chosen according to relevant factors such as its durability (the anodized NITUFF is used in military marine applications) and light weight. Pressure tests were performed to guarantee that the housing can withstand a pressure of 50 atmospheres, corresponding to a depth of 500 m.

**Figure 3:**
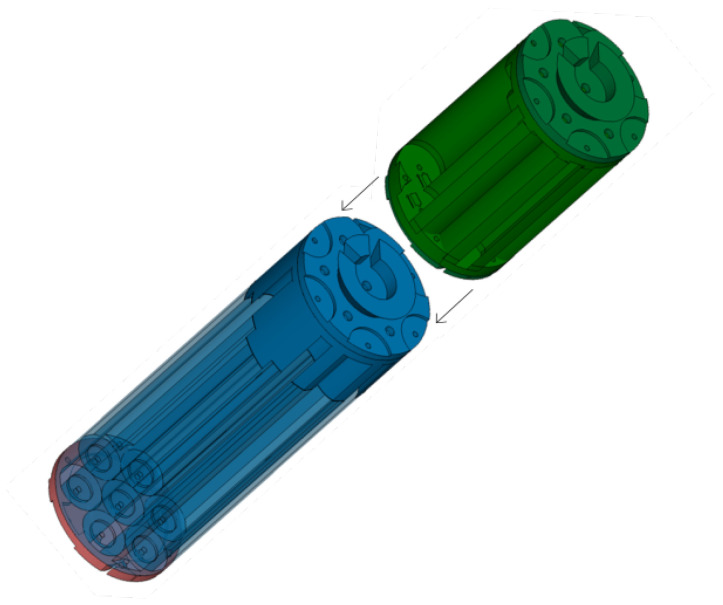
CAD Battery Blocks: top battery block (in green) corresponds to the 12 VDC preamplifier battery pack, bottom battery block (in blue) corresponds to the 6 VDC electronics.

Moreover, to maximize the energy density in the cylindrical housing, a hexagonal layout of D-cell batteries was created. The battery block to supply power to the electronic components is composed of 7 × 4 D-type batteries (see blue block in Figure 3). It should be noted that it was decided to use a voltage of 6 V instead of 4.5 V to take advantage of the length of the aluminum housing. Also, a shorter battery pack was designed with 8 D-type batteries placed in serial connection to provide 12 V to the C57 pre-amplified hydrophone (see green block in Figure 3). This hydrophone can be amplified in a range from 7.5 V to 30 V. We chose 12 V since this is a value that is maintained for deployments of 4 months.

#### 2.1.4. Ringed Buoys and Hydrophone Protection

Ringed buoys were used to enable the device to be handled and avoid acoustic shadows, providing the system with the necessary buoyancy and allowing deployments down to depths of 500 m. In addition, a removable cage was designed to mechanically hold the hydrophone in a vertical position, isolate it from vibrations and protect it from shocks. Table 2 shows a summary of the integrated functions.

### 2.2. Data Acquisition and Study Area

Data were collected using the previously described SAMARUC device from 16 May 2018 to 30 July 2018 and from 25 October to 25 November in the shallow waters of El Gorguel (37°33′42″ N, 0°53′14″ E) on the south-east coast of the Mediterranean Sea (Cartagena, Spain). This location allows the existing marine traffic in the Port of Cartagena to be monitored.

**Figure 4:**
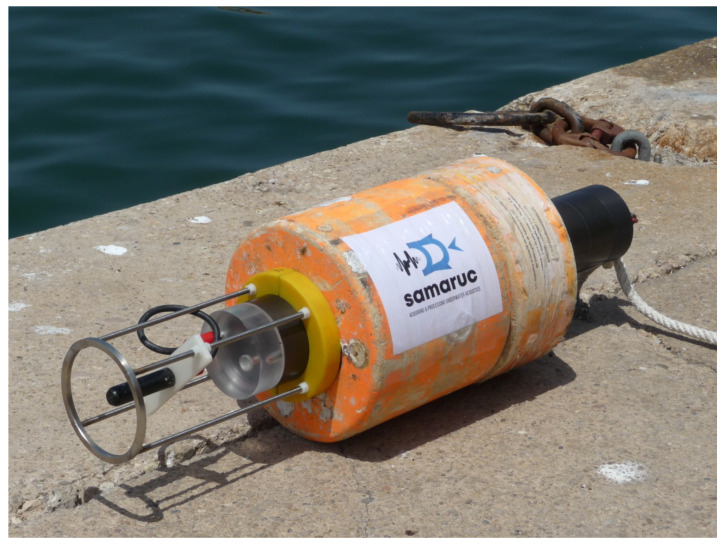
SAMARUC system.

The seabed in this area is not uniform and consists basically of mud. As described above, the system is prepared for 500 m depth deployments, but in this case, a depth of 60 m was chosen given the bathymetric profile of the exploration area. The device was placed 10 m from the seabed (reducing its acoustic influence) at a depth of 50 m using a 70 kg anchoring weight. The minimum distance between the device and the vessels’ permitted transit area was approximately 150 m (category B [1]). The sampling frequency was 192 kHz with a resolution of 16 bits and a device sensitivity set to −165 dB re 1 V/uPa (−167 dB re 1 V/uPa and hydrophone sensitivity of +2 dB of AIC3204 gain). The SAMARUC was configured with an operating cycle of 5 min ON and 10 min OFF. A picture of the entire SAMARUC system, including the ringed buoys used and the hydrophone protection cage, can be seen in Figure 4.

### 2.3. Numerical Modelling of Sound Propagation

The theoretical simulation of underwater noise due to ship traffic is a complex topic. Depending on the scenario to be considered, different models to calculate the propagation of acoustic noise through the medium are more appropriate than others. The integration of the parabolic equation [6], for example, gives good results for low frequency and shallow water environments. Normal modes and ray theory are also possible approaches to obtain numerical approximations of wave equation in a marine environment. As a result, it is important to study the different variables such as: depth of the seabed, the velocity profile of the water column, frequency to be studied, etc., when trying to establish the most appropriate modelling technique.

In this study, one more consideration was taken into account: the computation resources. In fact, this was the main reason the ray theory approximation (applying the public code BELLHOP [7]) was chosen instead of integration of the parabolic equation. One of the objectives associated with this work was to verify that the ray approximation provides sufficient accuracy if both receiver and emitter are at least half a wavelength away from the seabed or surface [8], since this method is more suitable for high frequency propagation.

**Figure 5:**
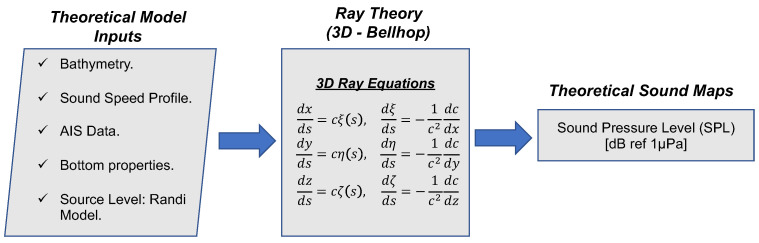
Flowchart for the computation of the sound maps.

Using the temperature, salinity, and depth/pressure data from the Argo profiler available on the EMODNET website [9], the sound velocity profile for the water column was calculated. In addition, marine traffic data was obtained through the AIS database provided for us. Bathymetry shapefile was defined using public data from the General Bathymetric Chart of the Oceans (GEBCO) [10]. The speed of sound versus depth was calculated by applying the Mackenzie equation [11] of nine terms due to its range of validity, considering a temperature of between 2 °C and 30 °C, a salinity of 25–40 parts per thousand (PPT) and a depth of between 0 m and 8000 m. The flowchart for the entire process is summarized in Figure 5.

One of the most important uncertainties in noise modelling is the source level assigned to each vessel, considered to be a noise emitter in the marine zone under study. Ships as an underwater acoustic source are a widely studied problem that remains open due to the complexity of the sound field emitted by each part of the ship at a given frequency. Several kinds of experimental models have been developed in recent decades, from the Ross model [12] that predicted the noise source level above 100 Hz considering the frequency and velocity of the ship, to the RANDI model that also took into account ship length.

Since the frequencies studied in this work are 63 Hz and 125 Hz, we chose the RANDI model to parameterize the source level of each ship considered in the computed AIS data. This method is validated for frequencies between 28.4 Hz–191.6 Hz. The mean value of the Sound Pressure Level (SPL) over the location was calculated considering the AIS data from vessels that have non-zero speed over ground.

### 2.4. Tracking Ships Using Acoustic Signatures of Engine Propeller Noise

Acoustic signatures of ship engine propeller noise can be used for noise characterization as well as for target tracking. Engine harmonics can be examined for full awareness of the effect of the different ships in the 1/3 octave ambient noise indicators. Additionally, vessel speed (*v*), closest point of approach (CPA), and draft can be estimated using a PAM system equipped with a single broadband hydrophone [13,14].

**Figure 6:**
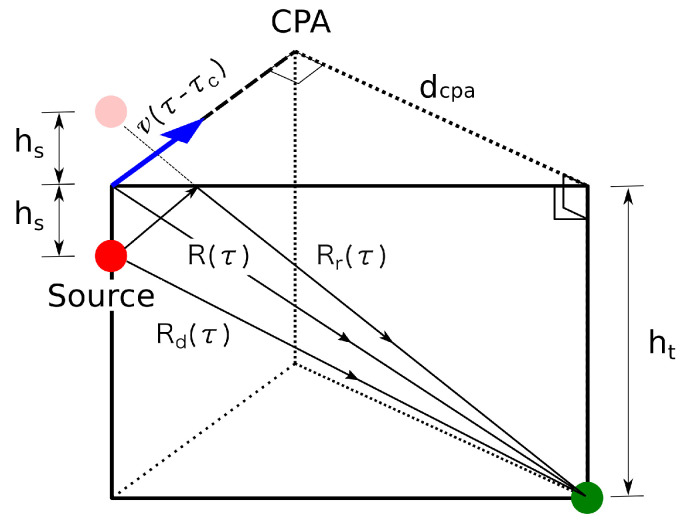
Lloyd’s mirror destructive-interference model.

A simple two-path ray model that only takes into account the direct contribution and the sea surface reflected gives quite accurate approximations, even in deep water [15]. As illustrated in Figure 6, the SAMARUC PAM system (green dot) is placed at depth $h_{t}$ and the source at depth $h_{s}$. The acoustic signal arrives at the sensor at time τ via a direct path $R_{d}\left(\tau \right)$, and via sea surface-reflected path $R_{r}\left(\tau \right)$. If the phases of these two main contributions cancel each other out, frequency destructive interference will occur. That happens when the phase difference between the two paths equals an odd multiple of π as shown in Equation (1).

(1) $$2\pi f_{n}\left(t\right)\frac{R_{r}\left(\tau \right)-R_{d}\left(\tau \right)}{c}=\left(2n-1\right)\pi$$

After some approximations [16,17], the equation to compute the $n^{th}$ frequency destructive interference at time *t* was obtained in Equation (A8) for the layout given in Figure 6. See Appendix A for details on the equation derivation.

(2) $$f_{n}\left(t\right)\approx \frac{2n-1}{4}\frac{c^{2}}{c^{2}-v^{2}}\frac{\sqrt{R_{c}^{2}\left(c^{2}-v^{2}\right)+c^{2}v^{2}{\left(t-\tau_{c}\right)}^{2}}-v^{2}\left(t-\tau_{c}\right)}{h_{s}h_{t}}$$

where $R_{c}=\sqrt{d_{cpa}^{2}+h_{t}^{2}}$, *c* is the sound speed in water, and $\tau_{c}$ is the time when the target is at the CPA. The frequency shift due to the Doppler effect can also obtained as in [16]. For a ship with an engine resonance at $f_{0}$, the Equation (A10) can be used to predict the frequency shift (see again Appendix A).

(3) $$f\left(t\right)=\frac{f_{0}c^{2}}{c^{2}-v^{2}}\left[1-\frac{v^{2}\left(t-\tau_{c}\right)}{\sqrt{R_{c}^{2}\left(c^{2}-v^{2}\right)+c^{2}v^{2}{\left(t-\tau_{c}\right)}^{2}}}\right]$$

## 3. Results and Discussion

**Figure 7:**
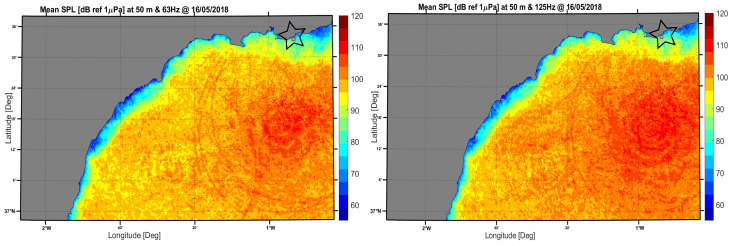
Sound map on 16/05/2018 at 63 Hz (**left**) and 125 Hz (**right**) calculated over the region studied.

The results obtained considering ray theory approximation are summarized in the sound maps of daily SPL dB re 1 uPa at a depth of 50 m. Figure 7 shows the daily sound map of the region studied after computing AIS data in the propagation model at frequencies of 63 Hz and 125 Hz on the day 16 May 2018. In addition, it is important to remark that both sound maps were calculated at a depth of 50 m, which is the depth where the SAMARUC device was installed (marked with a black star).

Daily sound maps of Figure 7 were calculated using four samples of AIS data for certain hours of the day (6:00 h–7:00 h, 14:00 h–15:00 h, 19:00 h–20:00 h and 23:00 h–00:00 h) at the same location of the SAMARUC, obtaining a daily mean value of pressure within a standard deviation interval (see Table 3).

Going back to real data obtained by SAMARUC, we calculated the average one-third-octave band (also in SPL dB re 1 uPa for 63 Hz and 125 Hz) for an integration interval of one hour at the same timestamp times of the modelling calculations. As Table 3 shows, both the one-third-octave band indicators from the real and theoretical data were calculated following the framework of Descriptor 11 [18] of the European MSFD.

The results show a good agreement between experimental and theoretical calculations, verifying not only that the device worked properly and the numerical simulations were valid, but also that the calibration of the system performed well at IEO-UPV’s facilities, an aspect not easy to achieve due to the low frequency range considered.

**Figure 8:**
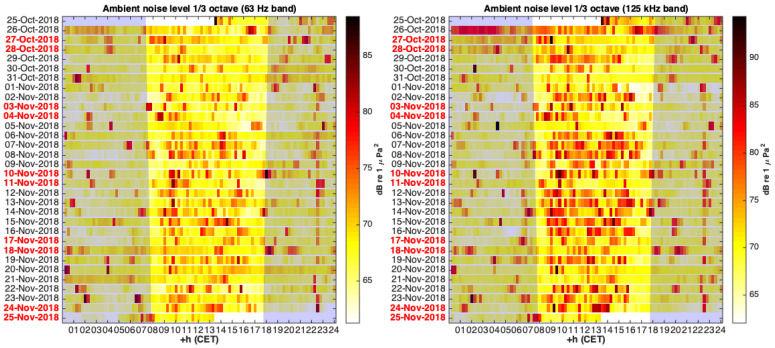
Heat map representation of 1/3 octave noise indicators in El Gorguel (Cartagena). Each pixel gives an indication of the noise level in a 15-min period. The left panel shows the $SPL_{1/3}$ at 63 Hz whereas the right panel shows the $SPL_{1/3}$ at 125 Hz. Sunrise/sunset times were calculated for longitude, latitude, and all deployment dates using a generic astronomy calculator. These sunrise/sunset times have been indicated using light shadow areas.

In addition to the aforementioned results, the computed 1/3 octave indicators can be conveniently structured in hours and days, and represented as a 2D color map to provide an acoustic panorama and identify seasonal events [19]. This representation technique is convenient when large temporal series need to be represented, as often happens with 1/3 octave ambient noise indicators. Figure 8 shows one of these representations for El Gorguel (Cartagena), where a repetitive acoustic event starting on 1 November 2018 at approximately 22:30 h can be detected. It also shows, as one might expect, higher ship noise levels in the 125 Hz band during the day than during the night.

**Figure 9:**
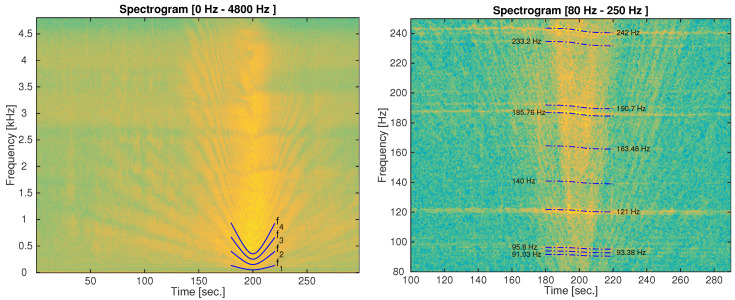
Spectrogram of a passing vessel in El Gorguel (Cartagena) and some of its corresponding frequency destructive-interference curves (**left**) as well as the Doppler shift curves (**right**). Spectrogram settings: FFT 1024 for the left spectrogram and FFT 16384 for the right spectrogram, Hamming window and overlap of 80%.

Let us now select one of the recordings containing a ship passing within the range of the SAMARUC system. The spectrogram for these real signals is shown in the left panel of Figure 9, and it is easy to see the U-shaped patterns due to the destructive interference varying with time. A magnified region of this spectrogram is shown in the right panel of Figure 9 where the Doppler effect can be measured. The fundamental frequencies of the ship engine are estimated using a time-frequency peak detector. Specifically, for the example shown, $f_{0}=\left[91.03,\;93.38,\;95.8,\;121,\;140,\;163.48,\;185.76,\;190.7,\;233.2,\;242\right]$ Hz.

The U-shaped structure and Doppler shift can be used to estimate the values for $R_{c}$, *v*, and $h_{s}$ ($h_{t}$ is the deployment depth and it is known) using the least squares method. The Equations (A8) and (A10) are used for this purpose, whereas only the first 4 interference curves were employed. The predicted frequency destructive-interference lines as well as the predicted Doppler shift lines are plotted overlaying the spectrogram and shown in Figure 9. It can be seen that the lines fit very well into the light curves of the spectrogram, thus validating the model.

## 4. Conclusions

In this work, we showed how anthropogenic continuous noise can be assessed in the context of the EU Marine Strategy Framework Directive. We started by designing and building a passive acoustic monitoring system and gave details that demonstrate how the system surpasses the required performance recommended for these devices. With the acoustic campaign performed in El Gorguel (Cartagena, Spain) as an example, we have illustrated how the theoretical sound maps can be created using marine traffic information, and how the real data can be used to experimentally validate the estimated sound pressure levels associated with continuous noise coming from anthropogenic activities. The system designed efficiently managed the power consumption and thus it is appropriate for long deployments. This fact allowed 2D ambient noise maps to be created, which could be used to identify seasonal events and to characterize ships by means of their acoustic signatures.

## Figures and Tables

**Table 1:** Specifications.

**Sensitivity of the pre-amplified hydrophone**	−167 dB re 1 V/uPa (Cetacean Research C57)
**# batteries used (with/without preamplification)**	36/28 (type D)
**AIC3204 programmable gain**	0–30 dB
**Storage capacity**	Currenty: (1 + 1) Tbytes
**Sampling rate**	192 kHz
**System bandwidth ±3 dB**	10 Hz–96 kHz
**Maximum depth**	500 m. (Housing and buoys)
**Autonomy**	Up to 4 months

**Table 2:** Summary.

Element	Functionality
File system	Increase the system storage capacity to 1 TByte (presently)
Specific electronics	Isolate the grounds. Use a second microSD card (storage capacity × 2)
Housing	6082 aluminum, widely used in marine applications
Battery block	Maximize energy density according to the tube dimensions
Flotation buoys	Ease of handling and avoidance of acoustic shadows
Protection cage	Hydrophone protection

**Table 3:** dB SPL re 1 uPa comparison for 63 Hz and 125 Hz on 16/05/2018.

16 May 2018	63 Hz				
Hours	6:00–7:00 h	14:00–15:00 h	19:00–20:00 h	23:00–00:00 h	Average
SAMARUC	78 ± 13	85.3 ± 0.5	79.7 ± 0.7	83.1 ± 1.2	82 ± 6
Theoretical results	81 ± 3	91 ± 5	78 ± 3	84 ± 2	83 ± 5
	**125 Hz**				
Hours	6:00–7:00 h	14:00–15:00 h	19:00–20:00 h	23:00–00:00 h	Average
SAMARUC	85 ± 4	90 ± 2	83.8 ± 0.3	81 ± 2	85 ± 9
Theoretical results	83 ± 4	93 ± 3	81.4 ± 1.1	81 ± 4	85 ± 6

## Abbreviations

The following abbreviations are used in this manuscript:

TGNoise	Technical Group on Underwater Noise
PAM	Passive Acoustic Monitoring
MSFD	Marine Strategy Framework Directive
GES	Good Environmental Status
DSP	Digital Signal Processor
ADC	Analog to Digital Converter
EMC	Electromagnetic Compatibility
GEBCO	General Bathymetric Chart of the Oceans
PPT	Parts per Thousand
AIS	Automatic Identification System
SPL	Sound Pressure Level
V	Volts
CPA	Closest Point of Approach

## Appendix A

To calculate the destructive-interference frequencies at a given time *t*, the source is assumed to be fixed at the position at an earlier time τ (τ < t). Let $R_{d}\left(\tau \right)$ and $R_{r}\left(\tau \right)$ be the lengths of the direct and surface-reflected paths respectively at time τ. From the Figure 6 we obtain:

$$\begin{array}{c} R_{d}\left(\tau \right)=\sqrt{v^{2}{\left(\tau -\tau_{c}\right)}^{2}+d_{cpa}^{2}+{\left(h_{t}-h_{s}\right)}^{2}}\\ R_{r}\left(\tau \right)=\sqrt{v^{2}{\left(\tau -\tau_{c}\right)}^{2}+d_{cpa}^{2}+{\left(h_{t}+h_{s}\right)}^{2}} \end{array}$$

Taking into account that the difference of two square roots can be written as $\sqrt{x}-\sqrt{y}=\left(x-y\right)/\left(\sqrt{x}+\sqrt{y}\right)$ and that $h_{t}\gg h_{s}$

(A1) $$R_{r}\left(\tau \right)-R_{d}\left(\tau \right)\approx 2h_{s}h_{t}/R\left(\tau \right)$$

where:

(A2) $$R\left(\tau \right)=\sqrt{v^{2}{\left(\tau -\tau_{c}\right)}^{2}+d_{cpa}^{2}+h_{t}^{2}}.$$

If the phases of the two main contributions cancel each other out, frequency destructive interference will occur. That happens when the phase difference between the two paths equals an odd multiple of π as shown in Equation (A3).

(A3) $$2\pi f_{n}\left(t\right)\frac{R_{r}\left(\tau \right)-R_{d}\left(\tau \right)}{c}=\left(2n-1\right)\pi$$

The *n*th frequency destructive interference at time *t* can be written as:

(A4) $$f_{n}\left(t\right)\approx \frac{2n-1}{4}\frac{cR(\tau )}{h_{s}h_{t}}$$

The relationship between *t* and τ is related to the time it takes the signal to propagate from the source to the receiver. In this way,

(A5) τ=t−R(τ)/c.

Substituting Equation (A2) into Equation (A5) and solving for τ gives:

(A6) $$\tau =\tau_{c}+\frac{c^{2}\left(t-\tau_{c}\right)-\sqrt{R_{c}^{2}\left(c^{2}-v^{2}\right)+c^{2}v^{2}{\left(t-\tau_{c}\right)}^{2}}}{c^{2}-v^{2}}$$

where $R_{c}=\sqrt{d_{cpa}^{2}+h_{t}^{2}}$. Using Equations (A5) and (A6) we can obtain:

(A7) $$R\left(\tau \right)=\frac{c}{c^{2}-v^{2}}\left[\sqrt{R_{c}^{2}\left(c^{2}-v^{2}\right)+c^{2}v^{2}{\left(t-\tau_{c}\right)}^{2}}-v^{2}\left(t-\tau_{c}\right)\right]$$

Finally, substituting Equation (A7) into Equation (A4) yields

(A8) $$f_{n}\left(t\right)\approx \frac{2n-1}{4}\frac{c^{2}}{c^{2}-v^{2}}\frac{\sqrt{R_{c}^{2}\left(c^{2}-v^{2}\right)+c^{2}v^{2}{\left(t-\tau_{c}\right)}^{2}}-v^{2}\left(t-\tau_{c}\right)}{h_{s}h_{t}}.$$

To obtain the frequency shift due to the Doppler effect we need to compute the instantaneous frequency, which is given by

(A9) $$f\left(t\right)=f_{0}\frac{d\tau }{dt},$$

where $f_{0}$ is the origin frequency. Using Equation (A6) in Equation (A9) and solving for *f* we obtain

(A10) $$f\left(t\right)=\frac{f_{0}c^{2}}{c^{2}-v^{2}}\left[1-\frac{v^{2}\left(t-\tau_{c}\right)}{\sqrt{R_{c}^{2}\left(c^{2}-v^{2}\right)+c^{2}v^{2}{\left(t-\tau_{c}\right)}^{2}}}\right].$$

That can be used to predict the frequency shift due to the Doppler effect.
